# Staged phalloplasty in a transgender male: a complex case report

**DOI:** 10.1093/jscr/rjag019

**Published:** 2026-01-30

**Authors:** Yasmine Ibrahim, Maxwell Zywica, Michael R DeLong

**Affiliations:** Division of Plastic and Reconstructive Surgery, University of California, Los Angeles, 200 Medical Plaza, Suite 460, Los Angeles, CA 90095, United States; Division of Plastic and Reconstructive Surgery, University of California, Los Angeles, 200 Medical Plaza, Suite 460, Los Angeles, CA 90095, United States; Division of Plastic and Reconstructive Surgery, University of California, Los Angeles, 200 Medical Plaza, Suite 460, Los Angeles, CA 90095, United States

**Keywords:** phalloplasty, gender-affirming care, genital reconstruction

## Abstract

We present a novel local groin flap approach for phalloplasty with minimal visible scarring. The presented patient is a 32-year-old transgender male with a prior metoidioplasty. He desired phalloplasty to optimized standing micturition and sexual function but was unwilling to undergo standard radial forearm or anterolateral thigh due to high visible scar burden. A staged approach to phalloplasty was employed, using a lower abdominal random pattern flap to construct the phallus. Once healed, future stages will create a urethral channel with full thickness skin graft from the thigh to maintain a low scar burden. Although this technique does not provide an option for direct neurotization of the construct, burial of the clitoral structures is expected to provide adequate sexual function. This technique can be considered for patients who do not desire the typical visible scarring with traditional phalloplasty methods.

## Introduction

Gender-affirming genital reconstruction for transgender men encompasses a variety of surgical procedures, with phalloplasty representing a principal technique. Phalloplasty involves reconstruction of the penis using various flap-based approaches historically developed for reconstruction following trauma or infection [1].

Various surgical approaches can create an anatomically representative phallus and functional urethra—allowing standing micturition while preserving erogenous sensation. However, complications such as urethral strictures and fistulae remain a concern [2].

The choice of phalloplasty technique is largely dictated by donor site selection, with commonly utilized sites including the radial forearm and anterolateral thigh (ALT) [1]. However, the extensive donor site morbidity can result in significant visible scarring and potential functional implications [3]. Therefore, surgical planning should consider donor tissue availability, anticipated donor site morbidity, and patient-specific goals.

This report presents a case of a transgender man who, more than a decade after metoidioplasty abroad, underwent a staged phalloplasty reconstruction beginning with a random pattern lower abdominal flap phalloplasty. A full thickness urethroplasty is planned as a subsequent stage and remains outstanding. This case illustrates the technical considerations of a secondary genital reconstruction and an alternative strategy to phalloplasty that minimizes donor site morbidity and operative duration.

## Case presentation

A 32-year-old transgender male presented with concerns regarding outcomes of a metoidioplasty following masculinizing hormone therapy performed overseas 14 years prior. His postoperative course was notable for a urethrocutaneous fistula, which developed 6 years after the initial surgery. An attempted repair failed within 2 months. Five years later, definitive fistula closure and bilateral testicular implants were placed; however, infection of the left implant required explantation.

At presentation, the patient reported persistent dissatisfaction with neophallus length, seeking revision primarily to improve standing micturition and sexual function. He expressed a preference for an abdominally based flap to minimize visible scarring and declined radial forearm, latissimus dorsi, or ALT phalloplasty. We then discussed the complexities of performing a vertical rectus abdominis myocutaneous flap versus a random pattern abdominal flap. On physical examination, he was noted to have thin, pliable abdominal tissue with minimal subcutaneous fat, which influenced flap selection and vascular reliability.

Given the limited availability of transposable tissue, single-stage reconstruction of the phallus and urethra was considered unlikely, and urethral reconstruction was planned for a later stage with full thickness skin graft to minimize visible donor site scarring. We then reviewed the limitations of an abdominal flap for phalloplasty in the initial stage, including unpredictable vascularity and lack of direct neurotization. The patient requested his existing metoidioplasty be preserved for potential later modification, understanding that this represents a deviation from standard phalloplasty protocols and entails trade-offs.

The patient ultimately opted for an abdominally based phalloplasty, declined skin grafting from his forearm or leg, and initiated electrolysis of the abdominal donor site.

**Figure 1:**
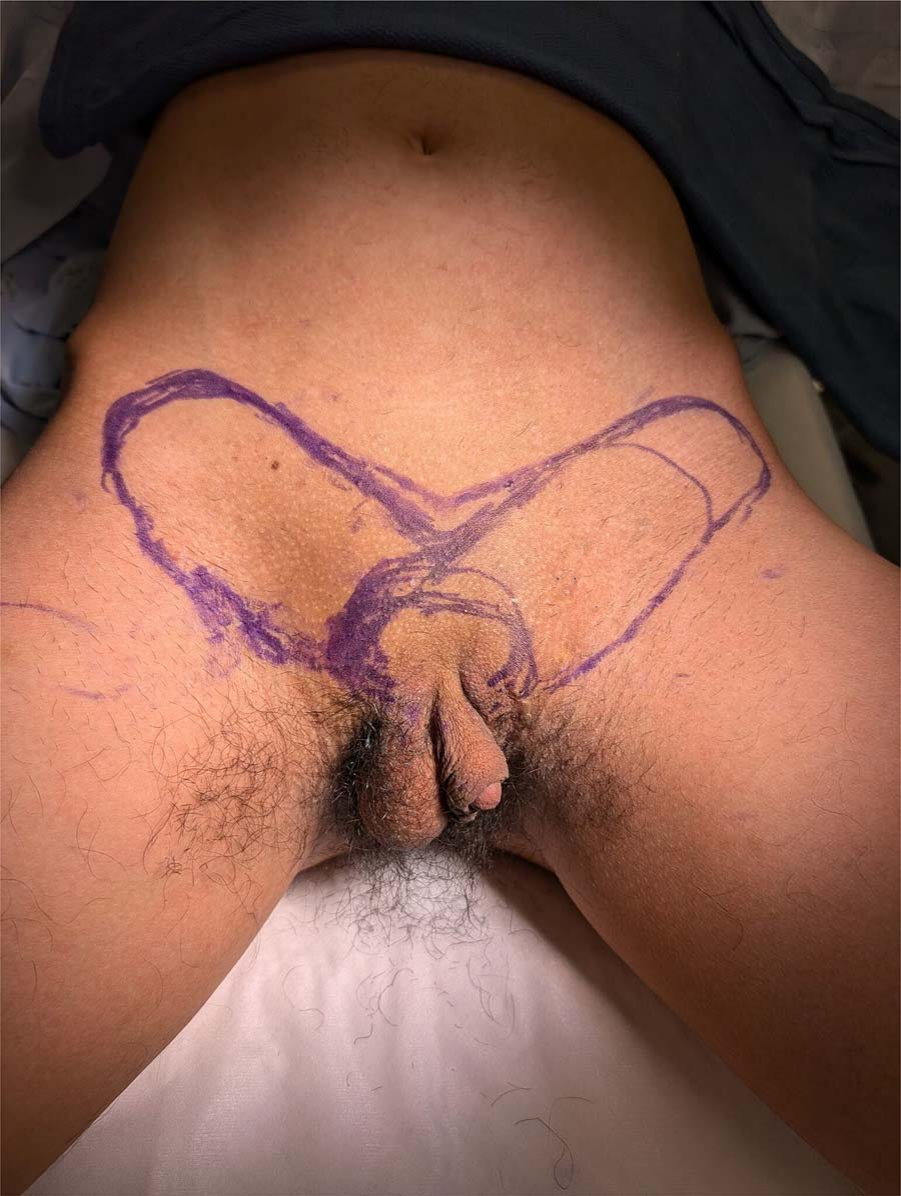
Preoperative abdominal flap markings.

With the patient’s consent, a staged reconstruction was undertaken, utilizing a lower abdominal random pattern flap for phallus creation. Bilateral flaps were designed as inferiorly based transposition flaps, each measuring on average ~6 cm in width by 12 cm in length, and intentionally oriented to conceal eventual scars (Fig. 1). Incisions were made with a scalpel, followed by cautery to identify the deep fascia. The flaps were elevated off the deep fascia, sparing all vessels at the base of the flaps. Flap rotation and advancement was periodically assessed intraoperatively. Once the flap could rotate into a phalloplasty and was bleeding from the tip, the phallus was tailor tacked into configuration.

**Figure 2:**
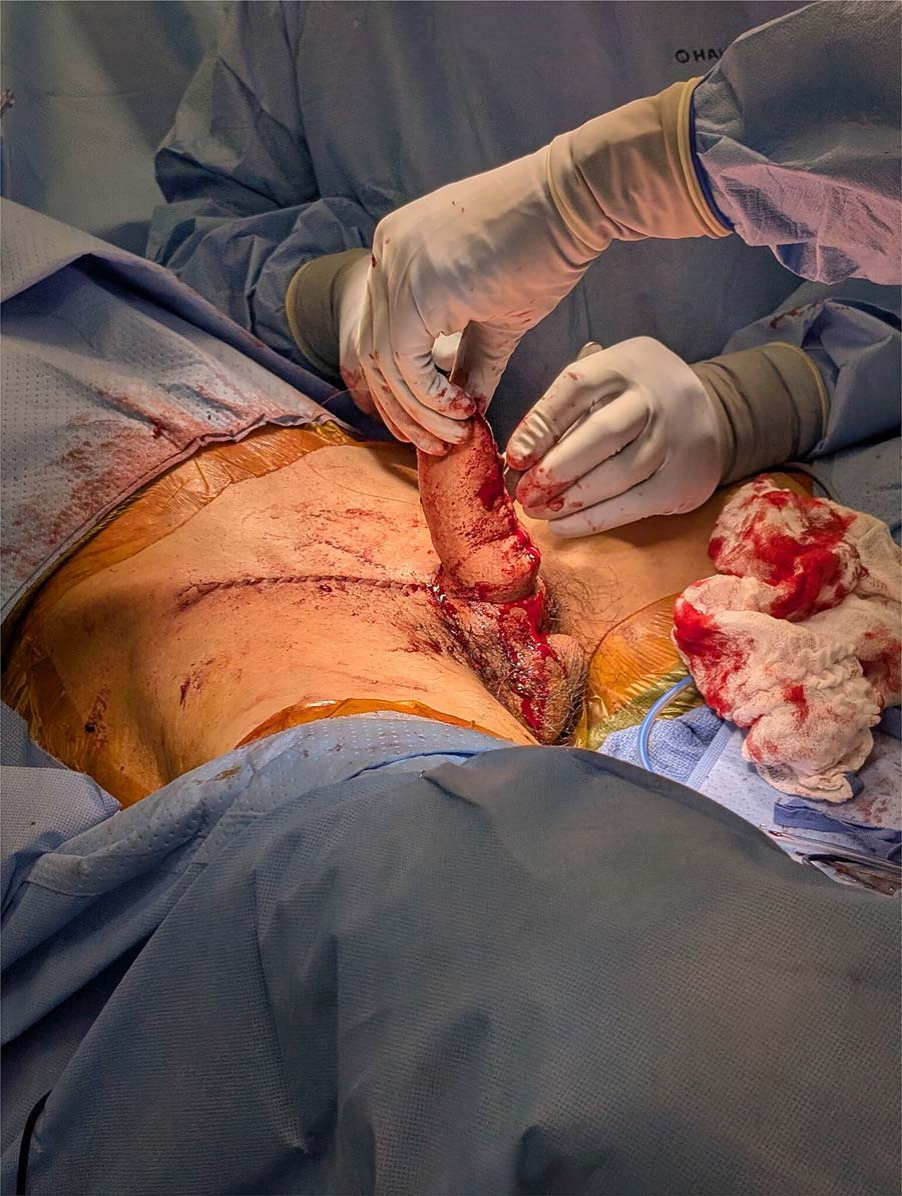
Intraoperative results.

The superior skin flap was elevated to facilitate tension-free closure. The wound was irrigated, and hemostasis was achieved with cautery. A 15 French drain was placed and secured with 2–0 nylon. Skin closure was completed with 3–0 and 4–0 Monocryl sutures in layered fashion (Fig. 2). The surgery was performed outpatient, and the operative duration was ~1–2 hours.

**Figure 3:**
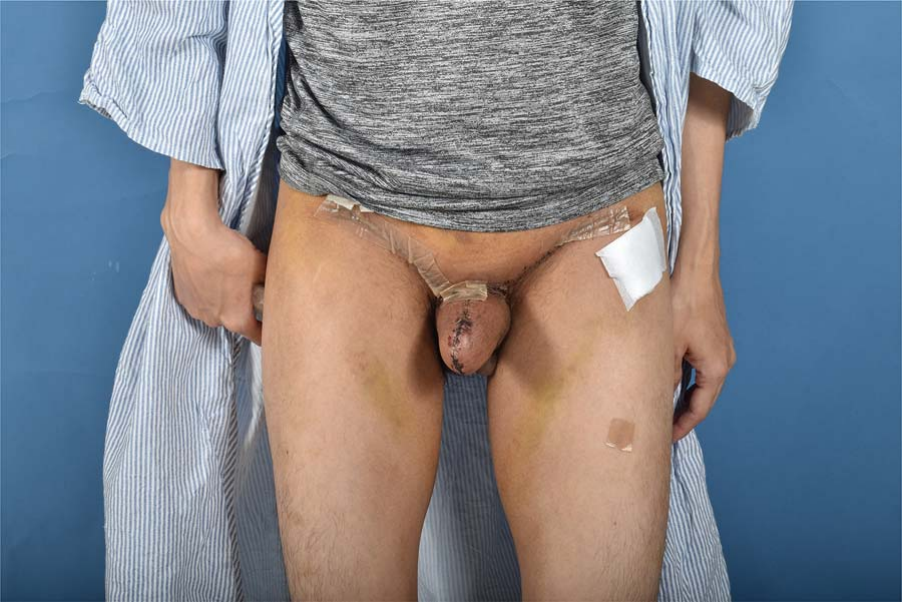
One month post-operative anterior view.

**Figure 4:**
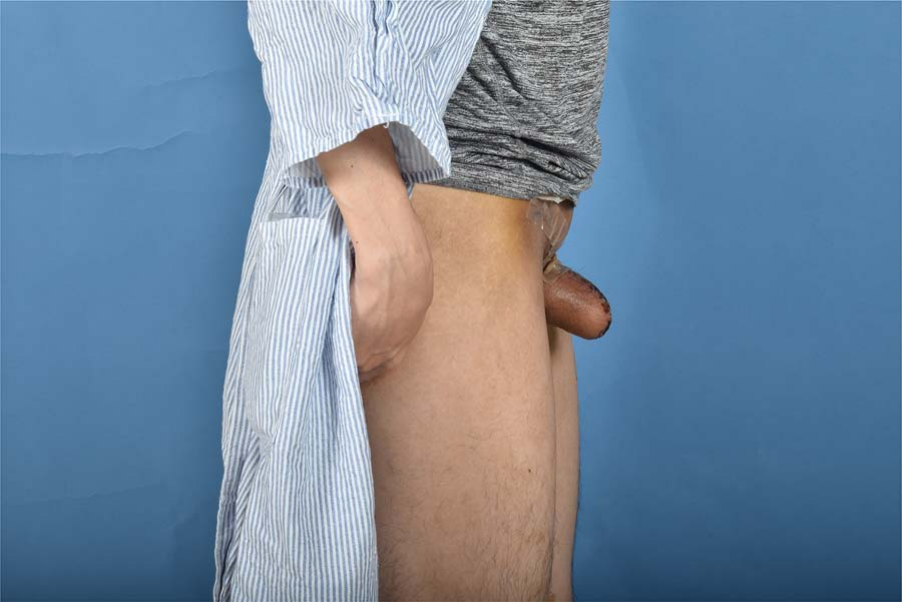
One month post-operative lateral view.

In postoperative follow up the full phallus remained viable without evidence of partial necrosis or incision dehiscence. The donor sites healed without incident and with very minimal linear scarring, well hidden in the bilateral groin creases. He is scheduled for future revision with full thickness skin graft placement for urethral reconstruction, which will eventually be connected to his current metoidioplasty (Figs 3 and 4).

## Discussion

This case demonstrated an approach to phalloplasty that prioritizes minimal, inconspicuous donor site scarring, and creates a viable foundation for future urethral creation. The multi-staged approach strategically employs the established use of skin grafting for future urethroplasty, aiming to minimize the patient’s risk of fistula recurrence [1, 4, 5].

The flap is categorized as a random pattern flap due to absence of large named vessels at its base. The superficial inferior epigastric and superficial circumflex arteries enter farther laterally. Preserving these vessels would limit adequate mobilization and shaping of the phallus, thus these vessels must be sacrificed. Skin paddles may be intentionally designed longer and then shortened intraoperatively using clinical exam or perfusion angiography. Although the absence of a named pedicle may reduce reliability, the technique avoids microsurgery, significantly reducing operative time and allowing completion as a 1–2-hour outpatient surgery.

Notable trade-offs of this approach include the absence of direct neurotization in the initial flap, limited neophallus length, and the need for multistage urethral reconstruction. Pressure applied directly on the neophallus is anticipated to provide sufficient stimulation for sexual function, although robust erogenous sensation is unlikely to develop.

Overall, this approach offers an alternative for patients who are not candidates for major free flap surgery or who wish to avoid the scarring and morbidity associated with radial forearm or ALT donor sites. Its limitations must be acknowledged, carefully considered, and clearly discussed with the patient when determining the optimal surgical strategy.

## Conclusion

This case highlights a staged approach to phalloplasty using an abdominal flap for phallus creation and a future full thickness skin graft urethroplasty, minimizing donor site morbidity and operative time while sacrificing initial neurotization. This strategy balances functional and aesthetic outcomes in complex secondary genital reconstruction. This option can be considered for appropriate patients who are not candidate for major free flap surgery or prioritize donor site scarring and morbidity.
